# Live Monitoring and Analysis of Fungal Growth, Viability, and Mycelial Morphology Using the IncuCyte NeuroTrack Processing Module

**DOI:** 10.1128/mBio.00673-19

**Published:** 2019-05-28

**Authors:** Sebastian Wurster, Pappanaicken R. Kumaresan, Nathaniel D. Albert, Paul J. Hauser, Russell E. Lewis, Dimitrios P. Kontoyiannis

**Affiliations:** a Department of Infectious Diseases, Infection Control and Employee Health, The University of Texas M.D. Anderson Cancer Center, Houston, Texas, USA; b Department of Pediatrics, The University of Texas M.D. Anderson Cancer Center, Houston, Texas, USA; c Clinic of Infectious Diseases, S’Orsola-Malpighi Hospital, Department of Medical and Surgical Sciences, University of Bologna, Bologna, Italy; Vallabhbhai Patel Chest Institute; HMH Center for Discovery and Innovation; Case Western Reserve University

**Keywords:** live imaging, antifungal treatment, fungal pathogens, morphogenesis, mycelium

## Abstract

Efficient live-imaging methods are pivotal to understand fungal morphogenesis, especially as it relates to interactions with host immune cells and mechanisms of antifungal drugs. Due to the notable similarities in growth patterns of neuronal cells and mycelial networks, we sought to repurpose the NeuroTrack (NT) processing module of the IncuCyte time-lapse microscopy system as a tool to quantify mycelial growth and branching of pathogenic fungi. We showed the robustness of NT analysis to study Candida albicans and five different molds and confirmed established characteristics of mycelial growth kinetics. We also documented high intra- and interassay reproducibility of the NT module for a spectrum of spore inocula and culture periods. Using GFP-expressing Aspergillus fumigatus and Rhizopus arrhizus, the feasibility of fluorescence-based NT analysis was validated. In addition, we performed proof-of-concept experiments of NT analysis for several translational applications such as studying the morphogenesis of a filamentation-defective C. albicans mutant, the effects of different classes of antifungals (polyenes, azoles, and echinocandins), and coculture with host immune cells. High accuracy was found, even at high immune cell-to-fungus ratios or in the presence of fungal debris. For antifungal efficacy studies, addition of a cytotoxicity dye further refined IncuCyte-based analysis, facilitating real-time determination of fungistatic and fungicidal activity in a single assay. Complementing conventional MIC-based assays, NT analysis is an appealing method to study fungal morphogenesis and viability in the context of antifungal compound screening and evaluation of novel immune therapeutics.

## Abstract (precis)

Pathogenic fungi remain a major cause of infectious complications in immunocompromised patients. Microscopic techniques are crucial for our understanding of fungal biology, host-pathogen interaction, and the pleiotropic effects of antifungal drugs on fungal cell growth and morphogenesis. Taking advantage of the morphological similarities of neuronal cell networks and mycelial growth patterns, we employed the IncuCyte time-lapse microscopy system and its NeuroTrack image analysis software package to study growth and branching of a variety of pathogenic yeasts and molds. Using optimized image processing definitions, we validated IncuCyte NeuroTrack analysis as a reliable and efficient tool for translational applications such as antifungal efficacy evaluation and coculture with host immune effector cells. Hence, the IncuCyte system and its NeuroTrack module provide an appealing platform for efficient *in vitro* studies of antifungal compounds and immunotherapeutic strategies in medical mycology.

## INTRODUCTION

Fungal morphology has been intensively studied due to its importance to the understanding of phenotypic plasticity (1) and host invasion by pathogenic fungi (2). Fungal morphogenesis is an important virulence trait affecting pathogenicity and shaping the innate immune response (3). Various environmental cues such as encounters with immune cells, mechanical impedance, or temperature shifts can induce hyphal branching (4). Inhibition of hyphal morphogenesis and filamentation has gained increasing attention as an antifungal drug target (5–9). In addition, morphological analyses in Candida albicans contributed to the dissection of mutations associated with nonsusceptibility to echinocandins and their fitness cost (10), as well as the effects of azoles on *Candida*’s invasive properties (11). Morphological features such as the number of active tips or branch points have been linked to metabolite yields in fermentation processes (12, 13), highlighting the relevance as a performance parameter for biotechnological applications (14, 15).

To facilitate morphological screening studies, simple, cost-effective, and robust live-imaging methods are needed. Whereas major improvements have been realized in terms of spatial resolution, (fluorescence) microscopy systems for real-time analysis are often low-throughput, and maintenance of *in vitro* conditions closely resembling the cells’ physiological environment remains a challenge (16, 17). The IncuCyte time-lapse fluorescence microscopy system, which is fully integrated in a cell culture or a microbiological incubator and able to analyze multiple culture vessels or well plates in parallel, overcomes many of these technical challenges (18, 19). A manufacturer-provided software package allows quantitative real-time analysis of cellular proliferation, viability, and morphology (18, 19). A number of novel protocols have been established to monitor chemotaxis, phagocytosis, and cell migration (20–22). Given the multitude of functional readouts, the IncuCyte system has attracted growing interest in the fields of immunology and infectious diseases (23–25), especially to study human immune cell interactions with a spectrum of pathogens (26, 27).

Unlike the majority of mammalian cell types, fungi are characterized by a highly heterogeneous appearance, significant phenotypic plasticity, and distinct morphological stages, ranging from single cells to polarized unbranched structures (germlings), to intricate filamentous networks (mycelium). This variability in growth patterns poses challenges to the automated assessment of fungal growth, requiring refined protocols capable of dissecting branched and interconnected mycelial networks in the setting of real-time imaging (12, 28). Recent work has highlighted the considerable similarities of mycelial and neuronal networks not only in terms of morphological characteristics but also in the governance of directional growth (29, 30). The need for reliable protocols to assay the formation of neuronal trees by polar extension and branching, a hallmark feature in neurobiology, has led to the development of the IncuCyte NeuroTrack (NT) image processing software module for automated real-time quantification of neuronal cell metrics such as neurite length, branch points, and cell clusters (31).

This study sought to repurpose the NT processing module for live analysis of fungal growth and hyphal branching. We evaluated, for the first time, the accuracy and reproducibility of optimized NT processing definitions to track mycelial expansion of six medically important fungi (yeasts and molds). We also provide proof-of-concept experiments for translational applications of IncuCyte NT analysis. Using a different major fungal pathogen for each application, we demonstrate the feasibility of NT analysis to study filamentation-defective phenotypes, antifungal efficacy, and coculture with host immune cells.

## RESULTS

### NeuroTrack image processing provides a reliable and reproducible tool to quantify growth and mycelial branching in a spectrum of human-pathogenic fungi.

**FIG 1:**
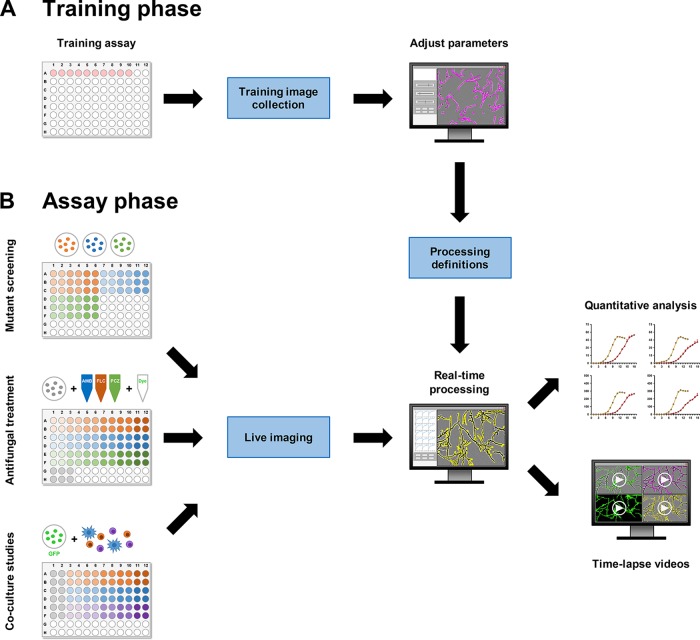
IncuCyte-based monitoring of fungal growth, branching, and viability. (A) Training phase. Representative images capturing different morphotypes of each fungus were used to compile training image collections. Analysis parameters were optimized for each fungus and processing module (Fig. S1). (B) Assay phase. Microplate assays, e.g., for fungal mutant analysis, antifungal treatment, or coculture with immune cells, were assembled in a 96-well format. GFP-expressing fungi or fluorescent dyes were used as needed. Phase ± fluorescence images were obtained in the IncuCyte Zoom time-lapse microscopy system. Processing definitions established in the training phase were used for real-time image processing, followed by statistical analysis and generation of time-lapse videos.

At first, we sought to qualitatively evaluate NT processing of phase-contrast images of C. albicans and five clinically relevant molds. Processing definitions were optimized individually for each tested species using representative training image collections (Fig. 1A). In the NT module, cell body segmentation was set to 0 (no detection of cell bodies) and the neurite width was adjusted to the expected hyphal width (2 μm or 4 μm). Subsequently, neurite sensitivity was gradually increased starting from 0.25 to obtain a setting completely detecting hyphal filaments in the focal plane but avoiding duplicate detection of the outlines of the same hyphal structure or false-positive masking (see Fig. S1A in the supplemental material). Once the optimal neurite sensitivity was determined, we reconfirmed that image processing was performed at the optimal neurite width by evaluating the alternative settings (Fig. S1A). The processing parameters for each fungal species are summarized in Table 1.

**TABLE 1:** Optimal processing parameters for phase-contrast images

Processing parameter	C. albicans	A. fumigatus	R. arrhizus	*R. pusillus*	*L. prolificans*	F. solani
NeuroTrack						
Segmentation mode	Brightness	Brightness	Brightness	Brightness	Brightness	Brightness
Segmentation adjustment	0	0	0	0	0	0
Neurite filtering	Best	Best	Best	Best	Best	Best
Neurite sensitivity	0.3–0.35	0.3–0.4	0.25–0.35	0.3–0.4	0.3–0.4	0.3–0.4
Neurite width (μm)	2	4	4	2	2	4
Basic Analyzer						
Segmentation adjustment	0.1	0.1	0.1	0.1	0.1	0.1
Adjust size	−1 or −2	−1 or −2	−1 or −2	−1 or −2	−1 or −2	−1 or −2

10.1128/mBio.00673-19.1 FIG S1 Establishment of processing definitions. The panels document the impact of key processing parameters on image masking by NeuroTrack (A) (A. fumigatus Af 293) and Basic Analyzer (B) (C. albicans Y4215) processing modules. Green frames and text boxes highlight the favorable settings. White arrowheads indicate mycelial filaments growing slantingly out of the focal plane. Their nondetection is not considered false-negative masking. Blue arrowheads indicate underdetection of hyphal filaments in the focal plane (false negative). Black arrowheads point to false-positive branch point masking, and red arrowheads indicate duplicate detection of the outlines of the same filament. Bar (both panels), 200 μm. Download FIG S1, TIF file, 5.2 MB. Copyright © 2019 Wurster et al. 2019 Wurster et al. This content is distributed under the terms of the Creative Commons Attribution 4.0 International license.

**FIG 2:**
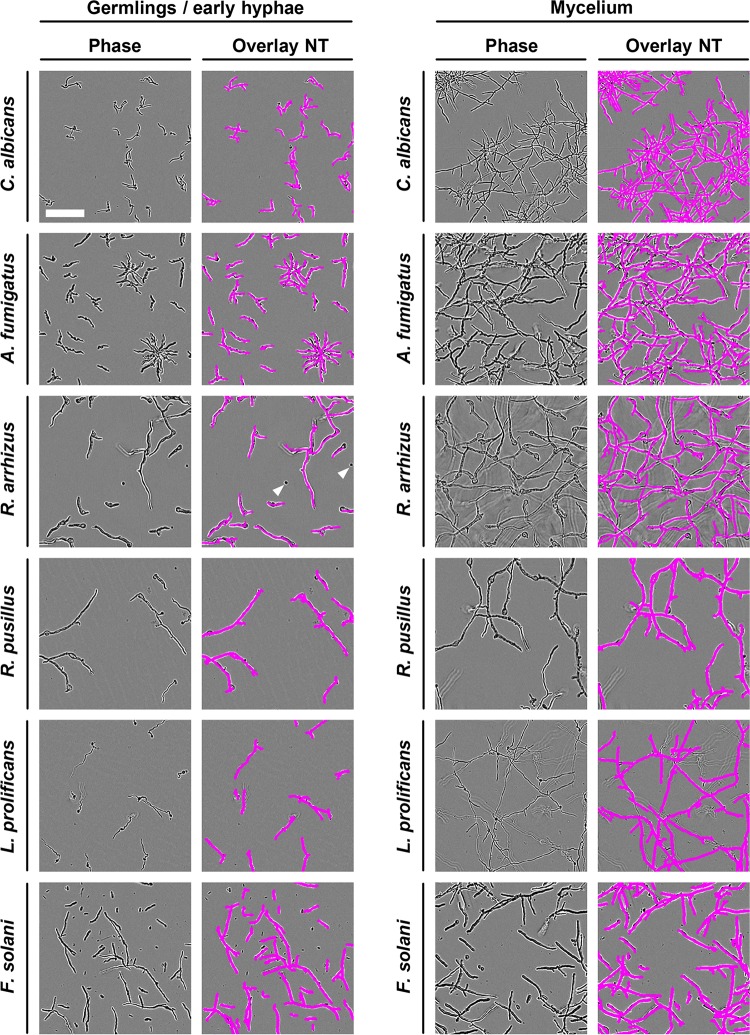
Representative images of NT processing definitions for six medically important fungi. Reference strains (C. albicans SC5314 and A. fumigatus Af 293) or clinical isolates (R. arrhizus strain 749, *R. pusillus* strain 449, *L. prolificans* strain 832, and F. solani strain 001) were seeded at 10^4^ spores per well of a 96-well flat-bottom plate in RPMI plus 2% glucose (+10% FCS for C. albicans). Phase-contrast imaging was performed hourly in the IncuCyte Zoom time-lapse microscopy system at 37°C for a period of 24 h (F. solani, 60 h). NeuroTrack (NT; pink) processing definitions were optimized for each individual fungus. Representative images of germlings/early hyphae and mycelium were selected. White arrowheads indicate examples of resting spores remaining unmasked by the NT module. Bar, 100 μm.

Despite considerable morphological variability among the tested fungal species, only minor differences in optimal processing settings were observed, suggesting that largely universal processing definitions can be established (Table 1). All NT processing definitions were highly accurate in the detection of both individual germlings or hyphal filaments and dense mycelial networks. Compared with the global confluence readout provided by the more widely available Basic Analyzer (BA) module (Fig. S1B and Fig. S2), NT processing yielded a much more accurate representation of mycelial networks (Fig. 2). In contrast, BA analysis struggled to resolve the individual structures and tended to mask areas framed by filaments (Fig. S2). This discrepancy between NT and BA processing was particularly prominent for C. albicans.

10.1128/mBio.00673-19.2 FIG S2 Representative examples of Basic Analyzer processing definitions for six medically important fungi. Reference strains (C. albicans SC5314 and A. fumigatus Af 293) or clinical isolates (R. arrhizus strain 749, *R. pusillus* strain 449, *L. prolificans* strain 832, and F. solani strain 001) were seeded at 10^4^ spores per well of a 96-well flat-bottom plate in RPMI plus 2% glucose (plus 10% FCS for C. albicans). Phase-contrast imaging was performed hourly in the IncuCyte Zoom time-lapse microscopy system at 37°C for a period of 24 h (F. solani, 60 h). Basic Analyzer (BA; blue) processing definitions were optimized for each individual fungus. Representative images of germlings/early hyphae and mycelium were selected. Bar, 100 μm. Download FIG S2, TIF file, 7.2 MB. Copyright © 2019 Wurster et al. 2019 Wurster et al. This content is distributed under the terms of the Creative Commons Attribution 4.0 International license.

**FIG 3:**
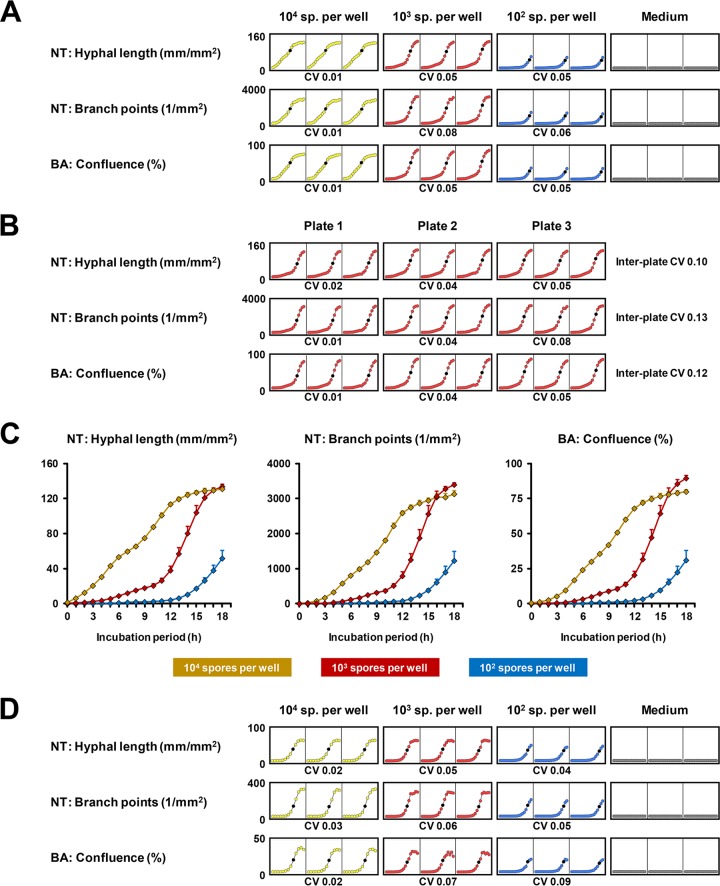
Intra- and interplate variability of Basic Analyzer and NeuroTrack processing definitions depending on fungal inoculum and culture period. (A to C) Spores of C. albicans reference strain SC5314 were plated in different concentrations (10^2^ to 10^4^ per well in 100 μl RPMI plus 2% glucose plus 10% FCS). Three plates, each containing triplicates of all tested concentrations, were prepared by different researchers to evaluate intra- and interplate reproducibility. Cell-free medium was used as background control (Medium). Phase images were obtained hourly for 18 h in the IncuCyte Zoom (37°C). Images were analyzed by Basic Analyzer (BA) and NeuroTrack (NT) processing modules. (A) Microplate graphs generated by the IncuCyte software are provided, showing technical triplicates on one representative plate. Coefficients of variation (CVs) were calculated at the time of the steepest incline in hyphal length (10^4^, 10 h; 10^3^, 14 h; 10^2^, 17 h), highlighted by a black dot in each diagram. (B) Microplate graphs comparing intra- and interplate variability of NT readouts and confluence for 10^3^ C. albicans spores per well. CVs were calculated based on 14-h values. (C) Comparison of NT and BA readouts depending on the C. albicans spore inoculum and culture period. Mean values based on 3 × 3 wells per condition and interplate standard deviations are shown. (D) The experimental setup described above (A to C) was applied to A. fumigatus reference strain Af 293. Results from one representative plate are shown. Intraplate CVs were calculated based on 8-h (10^4^), 12-h (10^3^), and 16-h (10^2^) values. Mean values and interplate variation are provided in Fig. S3.

In a second step, the reproducibility of NT and BA readouts was assessed using different inocula of selected fungi. For C. albicans Y4215, intra- and interplate variability of NT (hyphal length and branch points) and BA (confluence) parameters were monitored over a course of 18 h using 10^2^ to 10^4^ spores per well in a 96-well plate. Only the two higher inocula resulted in a confluence plateau by the end of the observation period. For all conditions, intraplate coefficients of variation (CVs) based on triplicate measurement were consistently below 0.1. Expectantly, higher fungal inocula tended to result in lower CVs (Fig. 3A). Interplate CVs were slightly higher but remained consistently below 0.2 (Fig. 3B). No significant difference in intra- or interassay CVs was found between the NT and BA readouts (Fig. 3B and C).

10.1128/mBio.00673-19.3 FIG S3 Interassay reproducibility of IncuCyte image analysis of mold pathogens. Different numbers of A. fumigatus Af 293, R. arrhizus strain 749, and F. solani strain 001 spores were dispensed in 96-well plates (10^2^ to 10^4^ per well in 100 μl RPMI plus 2% glucose). Three plates, each containing triplicates of all tested concentrations, were prepared by different researchers to evaluate interplate reproducibility. Phase images were obtained hourly for 18 h in the IncuCyte Zoom (37°C). Images were analyzed by NeuroTrack (NT) and Basic Analyzer (BA) processing modules. Mean values based on 3 × 3 wells per condition and interplate standard deviations are shown. The black triangle in the R. arrhizus BA panel indicates that, unlike NT analysis, the BA failed to continue to provide a reliable assessment for the 9- to 12-h time points. Download FIG S3, TIF file, 0.7 MB. Copyright © 2019 Wurster et al. 2019 Wurster et al. This content is distributed under the terms of the Creative Commons Attribution 4.0 International license.

We used the Aspergillus fumigatus reference strain Af 293 and clinical isolates of Rhizopus arrhizus and Fusarium solani to evaluate the reproducibility of NT and BA analysis in molds. While intra-assay CVs remained mostly below 0.15 (Fig. 3D and data not shown), interplate variability of both NT and BA image processing showed greater dependency on inocula for molds than C. albicans (Fig. S3). At 10^2^ spores per well, interplate CVs in early log phase went up to about 0.3 (A. fumigatus and R. arrhizus) and 0.2 (F. solani), respectively, but subsequently declined to <0.15 in mid-log phase. Taken together, these data suggest that NT monitoring is feasible and reproducible for a spectrum of medically important fungi, with both intra- and interplate variability meeting commonly applied acceptance criteria for cell-based bioassays (32, 33).

### NeuroTrack analysis of GFP-expressing or fluorescently labeled fungi facilitates reliable tracking of fungal growth patterns in coculture studies.

**FIG 4:**
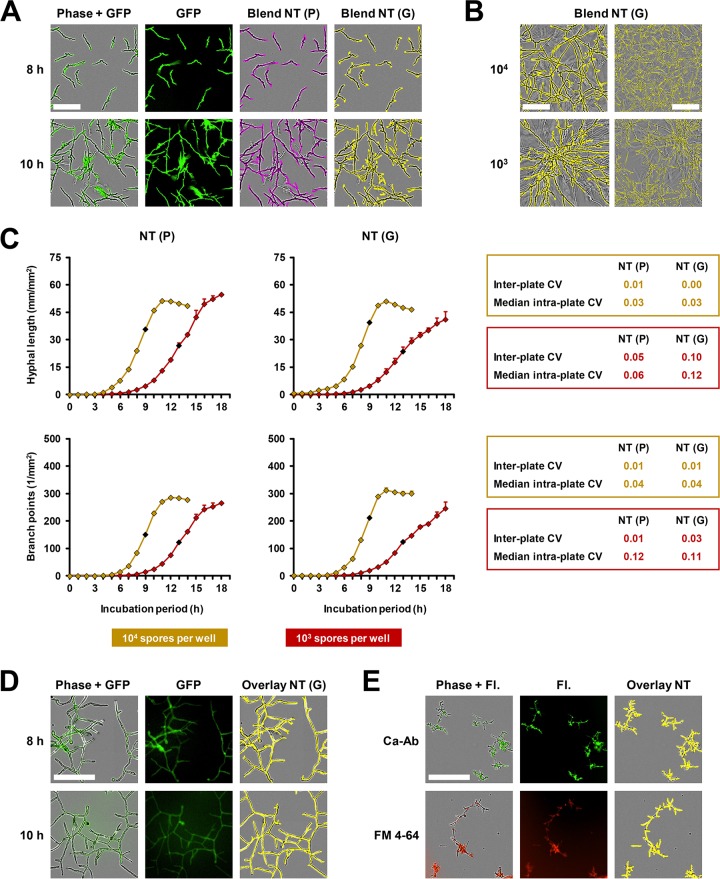
Fluorescence-based NeuroTrack analysis using GFP-expressing fungal strains or fluorescent staining. (A to C) Spores of a GFP-expressing variant of A. fumigatus reference strain Af 293 were seeded in a 96-well flat-bottom plate at a density of 10^3^ or 10^4^ per well in 100 μl RPMI plus 2% glucose. Three plates, each containing triplicates of both concentrations, were prepared by different researchers to evaluate intra- and interplate reproducibility. Phase and green fluorescence (acquisition time, 400 ms) images were obtained hourly for 18 h in the IncuCyte Zoom (37°C). Images were analyzed using phase-contrast-based [NT (P)] and green fluorescence-based [NT (G)] NeuroTrack processing definitions. (A) Representative images after 8 and 10 h of culture of 10^4^ spores per well are shown to document the accuracy of the processing modules in germlings and mycelium. Bar, 100 μm. (B) Representative images after 14 h of culture of both inocula are provided, documenting the accuracy of fluorescence-based NT analysis in complex mycelial networks including uneven growth patterns. Bars: left column, 100 μm; right column, 250 μm. (C) Comparison of NT readouts depending on the processing module, Af 293 spore inoculum, and culture period. Mean values based on 3 × 3 wells per condition and interplate standard deviations are shown. Key parameters comparing the two modules are summarized in the statistical insets next to the diagram. Coefficients of variation (CVs) were calculated at the time of the steepest incline in hyphal length (10^4^, 9 h; 10^3^, 13 h), highlighted by a black diamond in each curve. (D) Representative examples of an NT (G) processing definition applied to FTR1-GFP R. arrhizus after 8 and 10 h of culture (10^3^ spores in 100 μl RPMI plus 2% glucose). Bar, 200 μm. (E) Representative examples of fluorescence-based processing definitions applied to C. albicans reference strain SC5314 cultured for 8 h (10^3^ spores in 100 μl RPMI plus 10% FCS plus 2% glucose) and stained with 0.4% (vol/vol) antibody (Ab) 21164 (green) or 1% (vol/vol) FM 4-64 (red). Bar, 200 μm.

As certain applications such as coculture studies with host immune cells require fluorescent labeling of pathogens (21), we sought to evaluate the accuracy of fluorescence-based NT analysis. In the example of GFP-expressing A. fumigatus Af 293, we optimized and applied NT processing definitions to green fluorescence images [NT (G), Fig. 4A]. In both the germling and mycelium stages, excellent accuracy and high concordance with phase-based NT analysis [NT (P)] were seen. Highly accurate masking by the NT (G) algorithm was also confirmed in tight mycelial networks of GFP-Af 293 (Fig. 4B).

Quantitatively comparing the detection efficacy of GFP-Af 293 hyphae depending on the inoculum and processing method, we found highly comparable kinetics (*r* = 0.979 to 0.995, Lin’s coefficient of concordance ρ*_c_* = 0.922 to 0.990) of hyphal length and branching with either method (Fig. 4C; also Movie S1). The only notable difference, seen in two independent runs with 3 plates each, was a higher plateau for total hyphal length in NT (P) than in NT (G) at the 10^3^-spore inoculum (Fig. 4C and data not shown), probably attributable to the ability of NT (P) to follow hyphae growing out of the focal plane for a longer distance. However, NT (P) and NT (G) identified similar total hyphal lengths in mid-log phase, with G/P ratios of 1.10 and 0.88 for the 10^4^-spore and 10^3^-spore inocula, respectively. Similar to phase image processing, the higher inoculum (10^4^ spores) tended to provide smaller intra- and interplate coefficients of variation in mid-log phase, whereas no difference was seen between NT (P) and NT (G), indicating that phase- and fluorescence-based NT algorithms allow for comparably robust and reproducible analysis of fungal growth.

10.1128/mBio.00673-19.4 MOVIE S1 Phase- and fluorescence-based NeuroTrack analysis of GFP-expressing Aspergillus fumigatus Af 293. Spores of a GFP-expressing variant of A. fumigatus reference strain Af 293 were seeded in a 96-well flat-bottom plate at a density of 10^4^ per well in 100 μl RPMI plus 2% glucose. Phase and green fluorescence (acquisition time, 400 ms) images were obtained hourly for 12 h in the IncuCyte Zoom (37°C). Stacks of images with and without phase-based [NT (P), pink] and green fluorescence-based [NT (G), yellow] NeuroTrack masking were assembled to time-lapse videos as described in Materials and Methods. Download Movie S1, MOV file, 3.3 MB. Copyright © 2019 Wurster et al. 2019 Wurster et al. This content is distributed under the terms of the Creative Commons Attribution 4.0 International license.

To further corroborate the feasibility of fluorescence-based NT analysis, we qualitatively evaluated NT (G) masking of images of an FTR1-GFP R. arrhizus isolate (Fig. 4D) as well as C. albicans stained with a FITC-tagged-antibody (anti-C. albicans; Abcam; catalog no. 21164) or the red fluorescent yeast vacuolar membrane dye FM 4-64 (Sigma) (Fig. 4E). Though none of these labeling/staining strategies reached the long-lasting brightness and high signal-to-noise ratio of the GFP-Af 293 isolate, the accuracy of fluorescence-based NT analysis remained high, and no distraction of the processing algorithm by nonspecific background fluorescence was observed.

**FIG 5:**
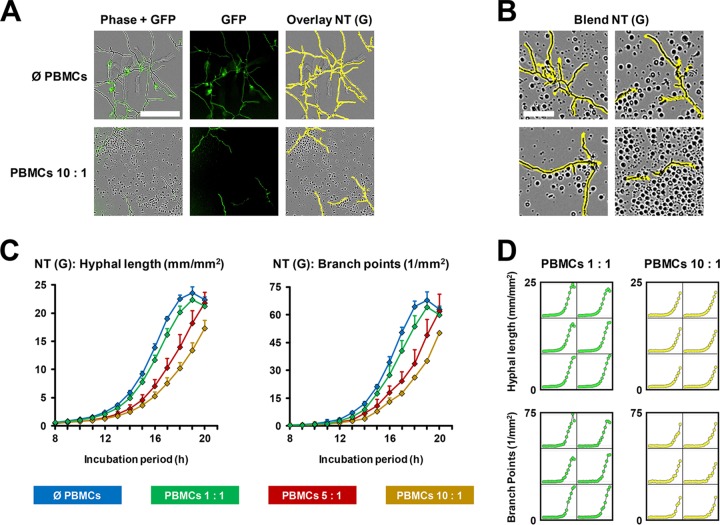
Monitoring of fungal growth and mycelial branching in mold-immune cell coculture experiments. GFP-expressing A. fumigatus reference strain Af 293, dissolved in 100 μl RPMI 1640 plus 10% FCS, was seeded/dispensed at 10e3 spores per well of a 96-well flat bottom plate. PBMCs from a healthy adult donor, suspended in 100 μl RPMI 1640 plus 10% FCS, were added at a ratio of 1, 5, or 10 to 1. Cell-free medium was added to the fungus-only control (Ø PBMCs). A PBMC-only control was used to detect unspecific background fluorescence (hyphal length of <0.1 mm/mm^2^ after 20 h; data not shown). Six wells were plated for each condition. The plate was incubated in the IncuCyte Zoom for 20 h (37°C). Phase and green fluorescence (400-ms acquisition time) images were obtained hourly. (A) Representative images of mycelial growth in the presence or absence of PBMCs are provided, and mycelial recognition by a GFP-based processing definition (yellow overlay) is shown. Bar, 200 μm. (B) Representative high-magnification images (bar, 50 μm), blended with the GFP-based NT processing module (yellow), are provided to document precise mycelium detection even in cases of immune cell accumulation in proximity to the fungus. (C) Hyphal length and branch point numbers were determined using a green fluorescence-based processing definition. Mean values and standard deviations are shown, covering a period from 8 to 20 h of culture (<1 mm/mm^2^ hyphal length and <1 branch point/mm^2^ for all conditions prior to 8 h of culture). (D) Microplate graphs generated by the IncuCyte software are provided, derived from the same experiment and covering the entire 20-h culture period.

Next, we tested the efficacy of NT (G) analysis in a coculture of GFP-Af 293 with human peripheral blood mononuclear cells (PBMCs). Detection accuracy remained unaltered in the presence of PBMCs (Fig. 5A). Even when immune cells accumulated in close proximity to fungal hyphae or fungal debris was encountered, near-optimal detection of hyphal filaments was achieved (representative examples are shown in Fig. 5B). In quantitative analysis of hyphal length and branching in PBMC-fungus cocultures, a delayed increase in both readout parameters was seen depending on the PBMC concentration, with an approximately 3-h delay at a 10:1 ratio (Fig. 5C; Movie S2). Though interwell variations were higher in the coculture setting than for fungus-only conditions, all CVs remained below 0.25, and most conditions and time points reached CVs below 0.1 (Fig. 5C and D).

10.1128/mBio.00673-19.5 MOVIE S2 NeuroTrack-based monitoring of growth and mycelial branching of Af 293 GFP-expressing A. fumigatus cocultured with PBMCs. GFP-expressing A. fumigatus reference strain Af 293, dissolved in 100 μl RPMI 1640 plus 10% FCS, was seeded at 10^3^ spores in each well of a 96-well flat-bottom plate. PBMCs from a healthy adult donor, suspended in 100 μl RPMI 1640 plus 10% FCS, were added at 10^3^ (1:1) or 10^4^ (10:1). Phase and green fluorescence (400-ms acquisition time) images were obtained hourly for 20 h. Stacks of images with and without green fluorescence-based NeuroTrack [NT (G), yellow] masking were assembled to time-lapse videos as described in Materials and Methods. Download Movie S2, MOV file, 5.0 MB. Copyright © 2019 Wurster et al. 2019 Wurster et al. This content is distributed under the terms of the Creative Commons Attribution 4.0 International license.

### NeuroTrack processing facilitates rapid and reliable assessment of antifungal efficacy and filamentation-defective fungal mutants.

**FIG 6:**
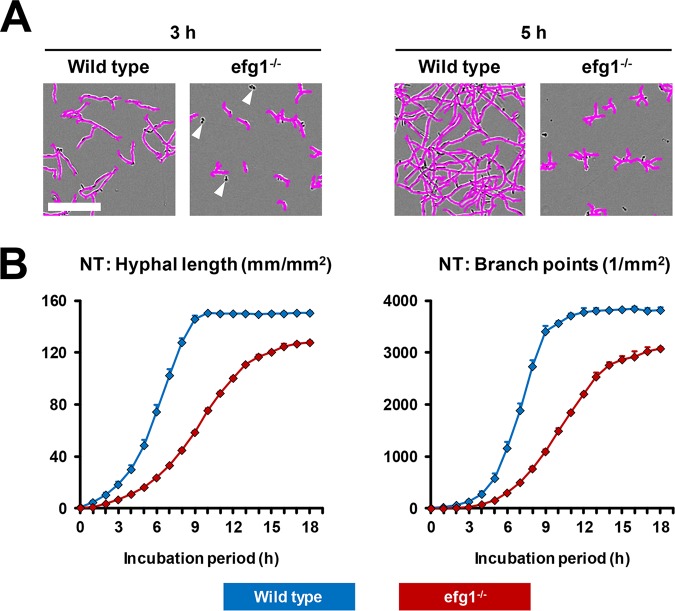
Assessment of a filamentation-aberrant C. albicans mutant by NeuroTrack analysis. Per well of a 96-well flat-bottom plate, 10^4^ spores of an *efg1*^−/−^ C. albicans mutant or its isogenic wild-type strain SC5314 were seeded in 100 μl RPMI plus 10% FCS. Phase imaging was performed hourly for 18 h in the IncuCyte Zoom. (A) Representative images after 3 h and 5 h of culture, overlaid by an NT processing definition, are shown. Bar, 100 μm. White arrowheads point to (correctly) nonmasked spores. (B) Hyphal length and branch point numbers were determined in a time course experiment. Mean values and standard deviations based on six wells per condition are provided.

To provide a proof of principle that NT analysis is able to recapitulate aberrant filamentation of a well-described C. albicans mutant, we compared NT analysis of a homozygous *efg1* null mutant (*efg1^−/−^*) and its isogenic wild-type strain SC5314. Representative images demonstrating the accurate NT-based recognition of the mutant’s reduced and protracted hyphae formation are provided in Fig. 6A. Both hyphal length and branch point numbers concordantly revealed a 6- to 8-h delay in hyphal growth and branching and lower plateau (Fig. 6B). The interwell variability of both readout parameters was consistently below 0.15 (Fig. 6B).

**FIG 7:**
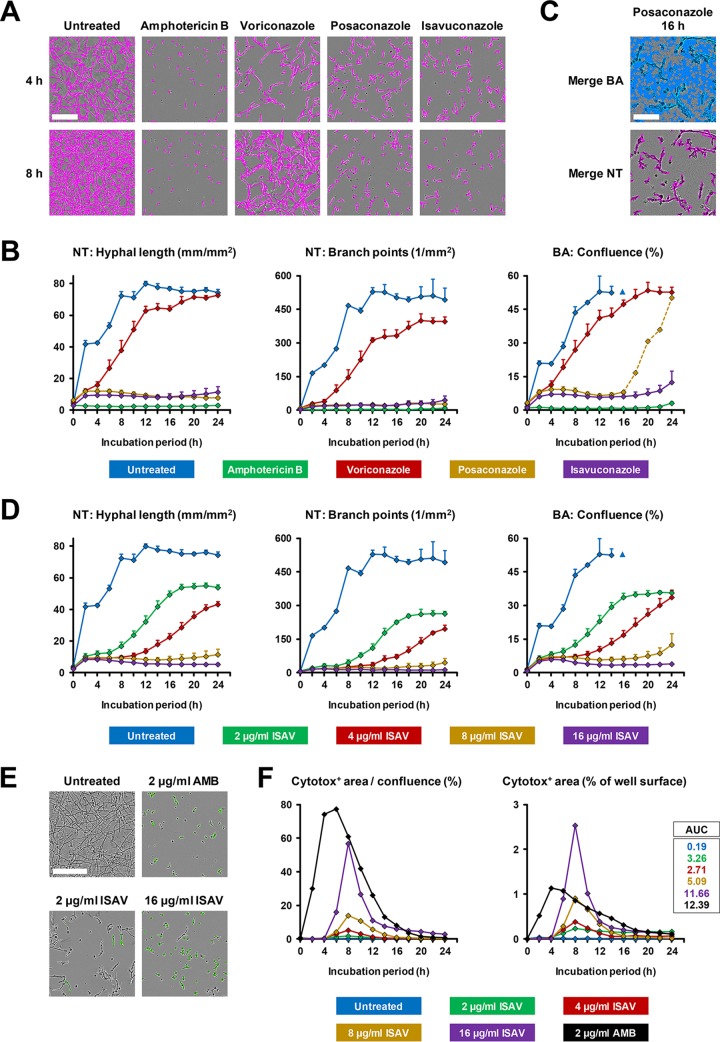
Impact of antifungal exposure on mycelial growth and fungal membrane integrity. (A to D) Per well of a 96-well flat-bottom plate, 2 × 10^3^ spores of R. arrhizus strain 749 (MICs: amphotericin B and posaconazole, 0.5 μg/ml; isavuconazole, 4 to 8 μg/ml; and voriconazole, 16 μg/ml) were seeded in 200 μl RPMI plus 2% glucose containing 2-fold serial dilutions of amphotericin B, voriconazole, posaconazole, or isavuconazole. All conditions were assessed in triplicates. The plate was incubated in the IncuCyte Zoom for 24 h (37°C), and phase images were obtained every 2 h. (A) Representative images, overlaid by an NT processing definition, after 4 and 8 h of culture in the presence of 8 μg/ml of each antifungal are shown. Bar, 200 μm. (B) Confluence, hyphal length, and branch point numbers were determined using BA (confluence) and NT (hyphal length and branch points) processing definitions. Mean values and standard deviations are provided depending on antifungal treatment and culture period. Due to extensive 3-dimensional growth, the BA module was not able to provide reliable results after the early plateau stage was reached (14 h, blue triangle). (C) Representative images showing the difference in accuracy of BA and NT analysis upon accumulation of fungal debris (16-h exposure to 16 μg/ml posaconazole). Bar, 100 μm. (D) Concentration-dependent inhibition of mycelial growth by isavuconazole (ISAV) was analyzed using BA and NT processing definitions. Mean values and standard definitions are given. (E and F) To determine the impact of antifungal exposure on fungal membrane integrity, 250 nM Cytotox Green dye was added to the culture setup described above. Phase and green fluorescence (400-ms acquisition time) images were obtained every 2 h for 24 h in the IncuCyte Zoom. (E) Representative images after 8 h of culture are provided. Bar, 200 μm. (F) The relative (green positive area/fungal confluence) and absolute (green positive area/well surface) Cytotox-positive areas were determined using phase- and green fluorescence-based BA processing definitions. The AUC (0 to 24 h) of the Cytotox-positive well surface was calculated with GraphPad Prism.

As antifungal drug discovery may be another important potential field of application, we performed NT analysis to document the inhibition of R. arrhizus growth and mycelial branching by amphotericin B and different triazoles (posaconazole, voriconazole, and isavuconazole). Representative examples of NT image analysis after 4 and 8 h of exposure to 8 μg/ml of each drug are shown in Fig. 7A. In line with MIC testing performed according to CLSI standards (results are provided in the figure legend), 8 μg/ml voriconazole, an azole that has very limited activity against Mucorales, led to a minor growth delay and lower branch point plateau, whereas all other antifungals substantially and sustainably inhibited mycelial growth (Fig. 7A and B), consistent with their activity against Mucorales. NT analysis remained undisturbed by accumulation of fungal debris, whereas false-positive detection of debris by the BA module was observed (e.g., for 8 and 16 μg/ml posaconazole, dashed line in Fig. 7B and representative example in Fig. 7C). In addition, the BA module was susceptible to minor imprecision of the autofocus upon onset of vertical mycelial growth (blue triangle in Fig. 7B and D). In contrast, both NT readouts showed a largely even plateau. In the example of isavuconazole, we confirmed that NT analysis is able to recapitulate a plausible dose-response correlation (Fig. 7D), with both NT readouts matching the visual MIC breakpoint determined according to the CLSI M38 broth microdilution reference method (data not shown).

Additionally, we used cytotoxicity staining (Cytotox Green; Sartorius) to test the fungicidal activity of isavuconazole in parallel to NT analysis. The Cytotox probe is inert and does not enter viable cells. As fungal membrane integrity diminishes, the probe enters the cell and fluorescently labels DNA. The green-fluorescence-positive area and total fungal area were quantified by BA processing. Minimal green positivity (Fig. 7E and F; Movie S3) and normal growth rates (data not shown) were seen in Cytotox-exposed untreated R. arrhizus, indicating that the probe is nontoxic at 250 nM. Peaking after 8 h, isavuconazole-exposed R. arrhizus exhibited a concentration-dependent increase in Cytotox positivity, with up to 55% of the cell area showing green fluorescence (Fig. 7E and F; Movie S3). Amphotericin B treatment was used as a positive control, causing a rapid peak in absolute and relative green fluorescent area.

10.1128/mBio.00673-19.6 MOVIE S3 NeuroTrack analysis and cytotoxicity staining of isavuconazole-exposed Rhizopus arrhizus. Spores of R. arrhizus strain 749 (2 × 10^3^) were seeded in 100 μl RPMI plus 2% glucose in a 96-well plate. Cytotox Green dye (250 nM; Sartorius) and 0 or16 μg/ml isavuconazole were added. Phase and green fluorescence (400-ms acquisition time) images were obtained at 37°C every 2 hours for 24 hours in the IncuCyte Zoom. Stacks of images with and without phase-based NeuroTrack [NT (P), pink] masking were assembled to time-lapse videos as described in Materials and Methods. Download Movie S3, MOV file, 5.4 MB. Copyright © 2019 Wurster et al. 2019 Wurster et al. This content is distributed under the terms of the Creative Commons Attribution 4.0 International license.

**FIG 8:**
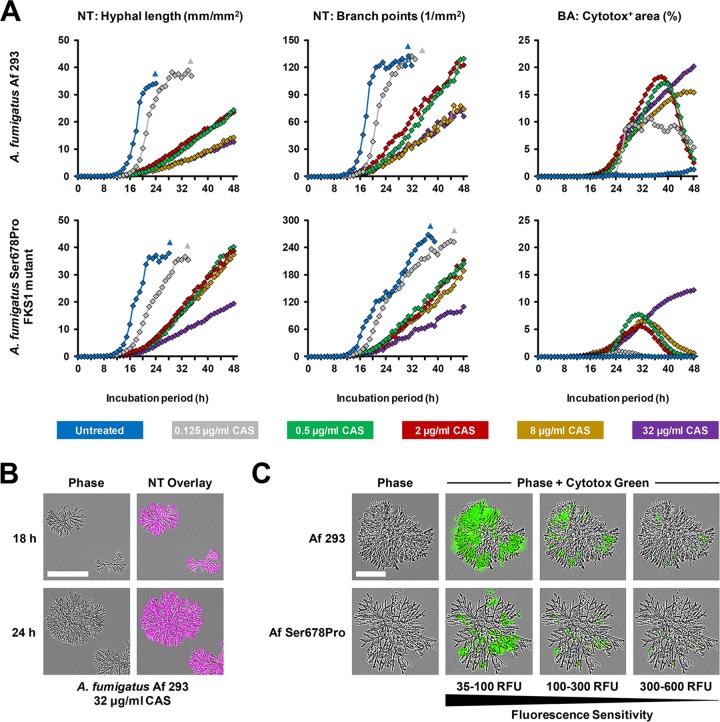
NeuroTrack analysis and cytotoxicity staining of caspofungin-exposed A. fumigatus. (A to C) Spores of A. fumigatus Af 293 or the echinocandin-resistant A. fumigatus FKS1 mutant strain Ser678Pro were seeded at 10^2^ per well of a 96-well flat-bottom in 200 μl RPMI plus 2% glucose supplemented with 0 to 32 μg/ml caspofungin (CAS). To determine the impact of CAS exposure on fungal membrane integrity, 250 nM Cytotox Green dye was added. Phase and green fluorescence images (400-ms acquisition time) were obtained hourly in the IncuCyte Zoom for 48 h (37°C). (A) Hyphal length and branch point numbers were determined using a previously established NeuroTrack (NT) processing definition. The Cytotox-positive area was determined using a green fluorescence-based Basic Analyzer processing definition. All conditions were assessed in triplicate. Mean values are shown. Coefficients of variation were consistently <0.25. Triangles indicate that analysis was terminated due to extensive vertical growth (diminishing precision of the autofocus). (B) Representative images of A. fumigatus Af 293 colonies after 18 and 24 h of culture in the presence of 32 μg/ml CAS are shown. Bar, 200 μm. (C) Representative phase and green fluorescence images of Cytotox Green-stained A. fumigatus Af 293 and Af Ser678Pro exposed to 32 μg/ml CAS are shown. Different fluorescence sensitivity settings were applied to document the degree of Cytotox positivity, with increasing RFU (relative fluorescence units) thresholds resulting in lower detection sensitivity. Bar, 100 μm.

Finally, we applied NT and Cytotox analysis to A. fumigatus exposed to serial dilutions of caspofungin (CAS), a drug known to induce significant morphological changes of the mycelial network and often incomplete growth inhibition in conventional assays such as broth microdilution-based minimum effective concentration (MEC) determination (34, 35). The echinocandin-susceptible reference strain Af 293 and a resistant FKS1 mutant strain (Af Ser678Pro) (36) were compared. In the absence of caspofungin, the two strains had similar hyphal length kinetics, reaching a plateau (in the focal plane) after 22 to 24 h, but the branch point kinetics of the mutant appeared to be protracted (Fig. 8A). For Af 293, CAS concentrations of 0.5 μg/ml or higher led to a significantly slower increase in total hyphal length, and a lower endpoint value was seen after 48 h, whereas the maximum hyphal length of the Af Ser678Pro mutant was reduced only by high concentrations of CAS (32 μg/ml). Accordingly, a markedly lower Cyotox-positive area was seen with the FKS1 mutant across all CAS concentrations tested. In contrast, the hyphal branch point endpoint largely failed to discriminate the wild-type and FKS1 mutant strains (Fig. 8A). The phenomenon of dissociated hyphal length and branch point kinetics of CAS-exposed A. fumigatus, attributable to the formation of highly branched rosettes with short hyphal segments as shown in Fig. 8B, recapitulates the well-described morphological alterations caused by echinocandins (34, 35). Evaluating cytotoxicity patterns at different fluorescence sensitivity settings, the highest affinity of the Cytotox probe was found at hyphal tips and recently developed subapical branch points (Fig. 8C), a finding that is in line with previously described fungal cell killing kinetics in echinocandin-exposed A. fumigatus (37).

Taken together, these proof-of-concept experiments suggest that NT analysis provides a reliable tool for monitoring filamentation-aberrant morphotypes and inhibition of mycelial expansion by exposure to immune effector cells and antifungal drugs. Further refinement by combining cytotoxicity staining and NT analysis allows for the parallel detection of inhibitory and fungicidal effects.

## DISCUSSION

Microscopic techniques are pivotal to our understanding of fungal morphology and physiology. Various image analysis systems have been developed to quantitatively assess mycelial characteristics (12), and the refinement of algorithms led to the establishment of fully automated classification schemes based on color micrographs (15) or fluorescence microscopy (2). The common growth pattern of neuronal cells and mycelia through branching and polar extension of filamentous cells (29) inspired the application of ImageJ’s (38) NeuronJ plugin to estimate the length of fungal hyphae (39).

While substantial progress in the field of microscopic imaging and image analysis algorithms significantly contributed to an improved understanding of fungal diversity, cell surface structures, regulation of fungal cell homeostasis, and host-pathogen interplay, there has been an unmet need for high-throughput real-time capture of cellular growth kinetics and morphological features (12). The recently introduced IncuCyte live-cell imaging system satisfies this need, integrating multiple imaging modes with customizable image processing algorithms. In the present study, we employed, for the first time, the system’s unique NT processing module to determine key mycelial growth parameters in medically important fungi.

To perform quantitative live-cell analysis in the IncuCyte software, processing definitions need to be tailored to the cell type or pathogen of interest through a user interface to account for specific morphologic features of the studied cell types or pathogens (31). Both the NT module and the more widely used BA are applicable to phase-contrast or fluorescent images, and analysis may be further refined by addition of viability, membrane integrity, and nuclear dyes (18, 19). Importantly, the NT module distinguishes cell bodies and neurites, allowing for the recognition and analysis of distinct morphological features (31). As we sought to provide a method for precise and reliable detection and quantitative analysis of mycelial networks, we adapted our processing definitions to ensure complete and accurate detection of neurites (hyphae). Specifically, to prevent recognition of dense mycelial clusters as a single cell body instead of distinct filaments, cell bodies were disregarded (segmentation adjustment = 0) while relatively low thresholds for neurite detection were used (Tables 1 and 2). We believe that these settings reflect an important difference between neuronal and mycelial morphology, the absence of prominent fungal cell bodies equivalent to the neuronal soma (29, 30). Reassuringly, formation of spherical pellets due to aggregation of mycelial biomass in liquid culture (e.g., fermentation processes) (12), the analysis of which may benefit from cell body recognition, was not encountered in our culture setting. However, if needed, simple adaptation of our processing definitions can be achieved by setting the cell body segmentation adjustment to 0.1 and applying an appropriate minimum cell body size to distinguish assemblies of branched networks from pellets.

**TABLE 2:** Optimal processing parameters for fluorescence-based NeuroTrack analysis

Parameter	C. albicans FM 4-64	C. albicans Ab 21164	A. fumigatus Af 293 GFP	R. arrhizus FTR1-GFP
Channel	Red^*a*^	Green^*b*^	Green^*b*^	Green^*b*^
Acquisition time (ms)	800	400	400	400
Color neurites				
Neurite coarse sensitivity	4–6	5–6	7–9	8–10
Neurite fine sensitivity	0.4–0.5	0.4–0.5	0.55–0.65	0.55–0.65
Neurite width (μm)	4	2/4	4	4

a Excitation wavelength, 585 nm; emission wavelength, 635 nm.

b Excitation wavelength, 460 nm; emission wavelength, 524 nm.

We thoroughly validated the NT approach for a range of medically important fungi. Despite major morphological differences, e.g., between the mycelial networks of Mucorales and C. albicans, this strategy led to a highly robust and reliable processing definition, requiring only minor adjustment for each individual pathogen (Table 1). In our experience, an important advantage of the NT processing module is the ability to adjust the preferably detected hyphal width (neurite width), further optimizing the accuracy of image analysis in the context of heterogeneous mycelial morphology. Hyphal length and branching kinetics seen in our experiments were in line with earlier studies (40, 41) which reported an initially exponential increase in total mycelial length and active tip formation (branch points). As described in a mathematical model for the hyphal growth unit by Steele and Trinci (42), a nearly linear correlation between total length and branch points per mm^2^ was found in the absence of immune cells or antifungals. As an additional means of validation, we demonstrated that NT analysis is able to recapitulate the filamentation-defective phenotype of the *efg1*^−/−^ strain, a well-described loss-of-function C. albicans mutant (43), suggesting a potential application of NT analysis in the screening of fungal mutant libraries.

Unlike previous efforts employing the oCelloScope platform for real-time monitoring of fungal inhibition and morphological changes (44), the IncuCyte technology allows for parallel fluorescence-based cytotoxicity readouts to evaluate both inhibitory and fungicidal effects in a single assay. This advantage is further enhanced by the system’s ability to image multiple 96-well or 384-well plates in parallel, rendering the IncuCyte a potential high-capacity platform for drug screening (45) and facilitating a streamlined evaluation of fungistatic and fungicidal drug efficacy, analogous to a protocol proposed for mammalian cells (46). To validate Cytotox staining in the context of antifungal drug exposure, we documented dose-response kinetics for different classes of antifungal agents and confirmed, in the example of CAS-treated A. fumigatus, that our assay resembles the cell lysis phenotype of apical cells and subapical branch points previously revealed by DiBAC staining (37).

In terms of translational applicability in medical mycology, our study revealed major advantages of NT-based image processing over the IncuCyte software’s more widely used BA module. Most importantly, the NT remained unbiased by fungal debris due to exposure to either antifungal drugs or immune cells (Fig. 7B). Additionally, the NT was less susceptible to minor inaccuracies of the autofocus upon onset of vertical growth than the BA and therefore provided an accurate reading for an extended period of time. Nonetheless, the BA has its place in the monitoring of fungal viability by cytotoxicity staining. As drugs eliciting early fungicidal activity prevent or at least significantly suppress germination and hyphal growth (Fig. 7E), NT analysis of Cytotox-stained fungus tended to underestimate the fungicidal effect (data not shown).

While the kinetics of the studied NT parameters of mycelial expansion were highly similar in untreated fungi, antifungal drug exposure led to a dissociation of hyphal length and branch point kinetics. In particular, A. fumigatus Af 293 exhibited much more prominent reduction in total hyphal length than branch point numbers when exposed to CAS, which matches the well-described multibudded rosette phenotype with shortened, slow-growing hyphal filaments (35). On the other hand, branch point quantification resulted in greater differences between untreated and triazole- or amphotericin B-exposed R. arrhizus. Therefore, it is crucial to elucidate the most relevant endpoints (e.g., hyphal length versus branch points), depending on the pathogen of interest and class of antifungal drugs. In addition, several reports highlight the impact of different culture periods on antifungal efficacy endpoints (47–49). Unlike most conventional microbiological methods, such as broth microdilution, XTT [2,3-bis-(2-methoxy-4-nitro-5-sulfophenyl)-2H-tetrazolium-5-caroxanilide salt] assays, or DiBAC staining, IncuCyte imaging allows for continuous monitoring of fungistatic and fungicidal parameters and thus streamlines the establishment of protocols to determine antifungal efficacy kinetics with excellent temporal resolution and a high level of objectivity.

It is important to recognize that there are further differences between IncuCyte image analysis and existing methods. Most conventional microbiological methods, such as visual MIC determination or XTT assays, rely on fungal biomass or global metabolic endpoints. Instead, IncuCyte image analysis could provide a rapid and convenient method to widen our understanding of how antifungals affect fungal morphogenetic programs. Further research and direct correlation with *in vivo* outcomes in animal models will be needed to define the comparative merits of the IncuCyte platform versus conventional microbiological methods to assess and predict the efficacy of antifungal drugs. As IncuCyte time-lapse microscopy is compatible with a broad selection of fungal growth media, our approach could also aid future studies focusing on the impact of therapeutic interventions on fungal growth and morphology in the setting of interkingdom infections or altered culture environments such as iron depletion (50) or changes in pH or O_2_ content.

Another major field that may benefit from NT analysis is the assessment of fungal cocultures with host immune cells, particularly in the context of increasing efforts to utilize primary or engineered (e.g., CAR-T cells) immune cells for therapeutic purposes in medical mycology (51–53). Fluorescence-based NT analysis facilitates highly reliable automated detection of germlings and mycelial filaments under conditions hampering phase-based segmentation such as coculture at high immune cell/fungus ratios. Thereby, our approach may aid the refinement and *in vitro* fungicidal efficacy validation of engineered cell therapeutics (P. R. Kumaresan et al., unpublished data).

Although IncuCyte imaging may help to explore these areas, limitations to NT studies of pathogenic fungi need to be considered. As the NT processing module operates strictly in two dimensions, NT analysis is poorly suited to track yeast biofilms, which are often subject to substantial vertical extension (11, 54). Similarly, although it provides reliable quantification of mycelial networks in the focal plane, IncuCyte imaging is not able to accurately represent mycelial biomass in advanced growth stages and thus may underestimate late-onset alterations to growth patterns, e.g., in the context of drug exposure. This is particularly relevant when studying Mucorales, characterized by early, sometimes explosive, formation of abundant mycelium (55). Therefore, it is crucial to assay a spectrum of spore inocula including seeding densities not resulting in rapid confluent growth (plateau stage) in order to monitor both early and protracted effects. Whereas our study documents acceptable intra- and interplate CVs for inocula as low as 100 spores per well, lowering spore inocula results in increased stochastic imprecision. Acquisition and analysis of multiple images per well, the use of the IncuCyte whole-well imaging module, or the use of larger well diameters (e.g., 24-well plates) may counteract fading precision at low spore concentrations but will reduce the system’s throughput and thereby set boundaries for large-scale screening approaches (56). For this reason, our study focused on the validation of setups with ≥100 spores per well. As another limitation, the NT method is not suitable to study yeasts that do not form hyphae or pseudohyphae under physiological conditions. For example, in the assessment of the emerging yeast pathogen Candida auris (57), IncuCyte analysis poorly correlated with established assays such as optical density or growth curves determined by manual counting (data not shown).

In summary, despite some limitations, the IncuCyte imaging system and its NT processing module provide an innovative and reliable tool to track growth patterns in the context of a vastly heterogeneous microscopic appearance and the existence of disparate morphotypes in human-pathogenic fungi. Our findings recapitulated key characteristics of mycelial growth, and a high degree of accuracy and reproducibility of NT readouts was documented for both phase-contrast and fluorescence imaging of several medically important fungi. Preparing the ground for translational applications, proof-of-concept experiments demonstrated the feasibility of NT analysis in the context of fungal mutant assessment, antifungal drug exposure, and immune cell encounters. Combined with the system’s high-throughput real-time imaging capabilities and its user-friendly interface requiring minimal training, NT analysis would be positioned as an appealing platform for large-scale *in vitro* screening of antifungal compounds or the preclinical evaluation of novel immune therapeutic strategies.

## MATERIALS AND METHODS

### Source and culture of fungal isolates.

Clinical isolates of Candida albicans (strain Y4215), Rhizopus arrhizus (strain 749), Rhizomucor pusillus (strain 449), Fusarium solani (strain 001), and Lomentospora prolificans (strain 832) were obtained from patients at The University of Texas M.D. Anderson Cancer Center, Houston, TX. In addition, the following reference strains and mutants were used: C. albicans SC5314, wild-type and GFP-expressing Aspergillus fumigatus Af 293, and A. fumigatus Af Ser678Pro (36); C. albicans *efg*^−/−^ (43); and FTR1-GFP R. arrhizus (58).

All C. albicans isolates were streaked on yeast peptone dextrose agar (YPD) plates for single-colony isolation. Single colonies were then grown in 5 ml of YPD liquid medium overnight at 35°C. On the following day, 100 μl of the overnight cultures was added to 5 ml of fresh YPD liquid medium and grown to mid-log phase. F. solani was grown in 5 ml of YPD liquid medium for 3 to 5 days at 30˚C. The FTR1-GFP R. arrhizus mutant was kept on YNB+csm (complete supplement mixture)-uracil medium for 3 to 5 days. All other tested molds were grown on yeast extract agar medium (YAG) for 48 to 72 h at 37°C and collected in saline by gently scraping the mycelium with a sterile glass rod. Fungal suspensions were washed twice with sterile saline, and spore concentrations were determined using a hemocytometer.

### Antifungal exposure and cytotoxicity assay.

Twofold serial dilutions of amphotericin B (Sigma-Aldrich), voriconazole (Sigma-Aldrich), posaconazole (Toronto Research Chemicals), isavuconazole (Toronto Research Chemicals), and caspofungin (Sigma) were prepared in 96-well flat-bottom plates (100 μl per well). R. arrhizus or A. fumigatus spores were diluted in RPMI plus 2% glucose at a concentration of 1 × 10^3^ to 2 × 10^4^/ml depending on the pathogen and drug (specified in the figure legends). For membrane integrity staining, Cytotox Green dye (Sartorius; final concentration, 250 nM) was added. One hundred microliters of the fungal suspension was added to each well. Each condition was assessed in triplicate.

### Isolation of PBMCs.

Informed consent was obtained from healthy adults (MDACC Institutional Review Board protocol LAB07-0296), and 20 ml EDTA-whole blood was collected. To isolate peripheral blood mononuclear cells (PBMCs), the blood was diluted 1:1 with phosphate-buffered saline (PBS), layered onto Ficoll-Paque Plus (GE Healthcare), and centrifuged at 400 × g for 30 min at room temperature (RT). Interphase cells were collected, washed twice with PBS, and resuspended at 10^5^ cells per ml in RPMI plus 10% fetal calf serum (FCS).

### Fungus-immune cell coculture experiments.

Fungal spores were diluted in RPMI plus 10% FCS at a concentration of 2 × 10^4^/ml. Fifty microliters (1 × 10^3^ spores) was seeded per well of a 96-well flat-bottom plate. Ten microliters (1 × 10^3^, 1:1 ratio), 50 μl (5 × 10^3^, 5:1 ratio), or 100 μl (1 × 10^4^, 10:1 ratio) of a 1 × 10^5^/ml PBMC suspension was added to the fungus-containing wells. The volume in each well was adjusted to 150 μl by addition of cell-free medium.

### Imaging and image analysis.

Well plates were imaged hourly or every 2 h in the IncuCyte Zoom HD/2CLR time-lapse microscopy system (Sartorius) (18) equipped with an IncuCyte Zoom 10× Plan Fluor objective (Sartorius). Imaging was performed for 16 to 60 h at 37°C. Imaging periods and intervals for each individual experiment are specified in the figure legends. Phase images were acquired for every experiment. For fluorescence imaging, the acquisition times were 400 ms for the green channel and 800 ms for the red channel. Representative images capturing different morphotypes and fungal cell densities were used for training image collections (Fig. 1A). Analysis parameters for Basic Analyzer (BA; endpoint, confluence [%]) and NT (IncuCyte Zoom NeuroTrack software module; endpoints, neurite length [mm/mm^2^] and branch points [1/mm^2^]) processing definitions were optimized individually for each species and (if applicable) fluorescent labeling strategy according to the workflow outlined in the manufacturer’s manual (31). Ranges of key processing parameters are summarized in Table 1 (phase) and Table 2 (fluorescence). The optimized processing definitions were subsequently used for real-time image analysis (Fig. 1B).

### Generation of time-lapse videos.

Time-lapse movies were generated by capturing phase and fluorescence images of fungal mycelia at 1-h intervals for at least 12 h. Stacks of images with and without NT processing definition overlays were exported in tagged image file format (TIF) using the time plot function in the IncuCyte graph/export menu. Videos were assembled in Microsoft PowerPoint, exported in MP4 format, and converted to MOV format.

### Statistics.

Microplate graphs were generated using the time plot feature in the graph/export menu of the IncuCyte Zoom software. Raw data for confluence, neurite (hyphal) length, branch point numbers, and fluorescence-positive area (if applicable) were exported to Microsoft Excel and GraphPad Prism to calculate mean values, measures of variability, coefficients of correlation and concordance, and area under the curve (AUC). Coefficients of variation (CVs) were calculated by dividing standard deviations by arithmetic means. FDA recommendations for bioanalytical method validation suggest a CV of 0.15 to 0.2 as an acceptance criterion (32), whereas a CV of 0.3 is considered acceptable for cell-based bioassays (33), to take into account an inevitable inoculum-dependent random error of the cell counting/cell seeding procedures (59).
