# Supplementary figures and images for: Live Monitoring and Analysis of Fungal Growth, Viability, and Mycelial Morphology Using the IncuCyte NeuroTrack Processing Module
Source: mBio. 2019 May 28;10(3):e00673-19. doi: 10.1128/mBio.00673-19 (PMC6538782; doi:10.1128/mBio.00673-19)

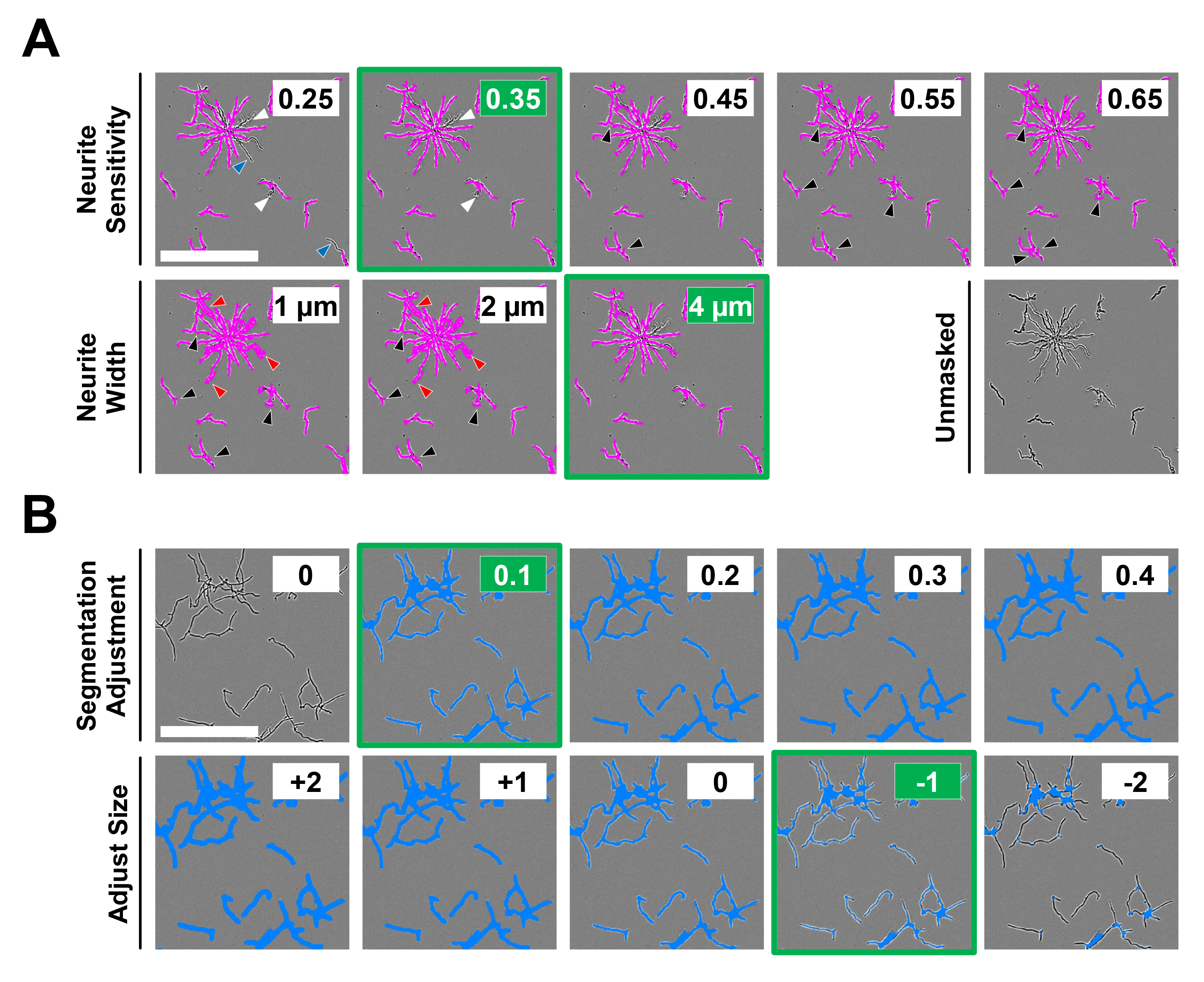

Supplement: FIG S1 [file mBio.00673-19-sf001.tif]

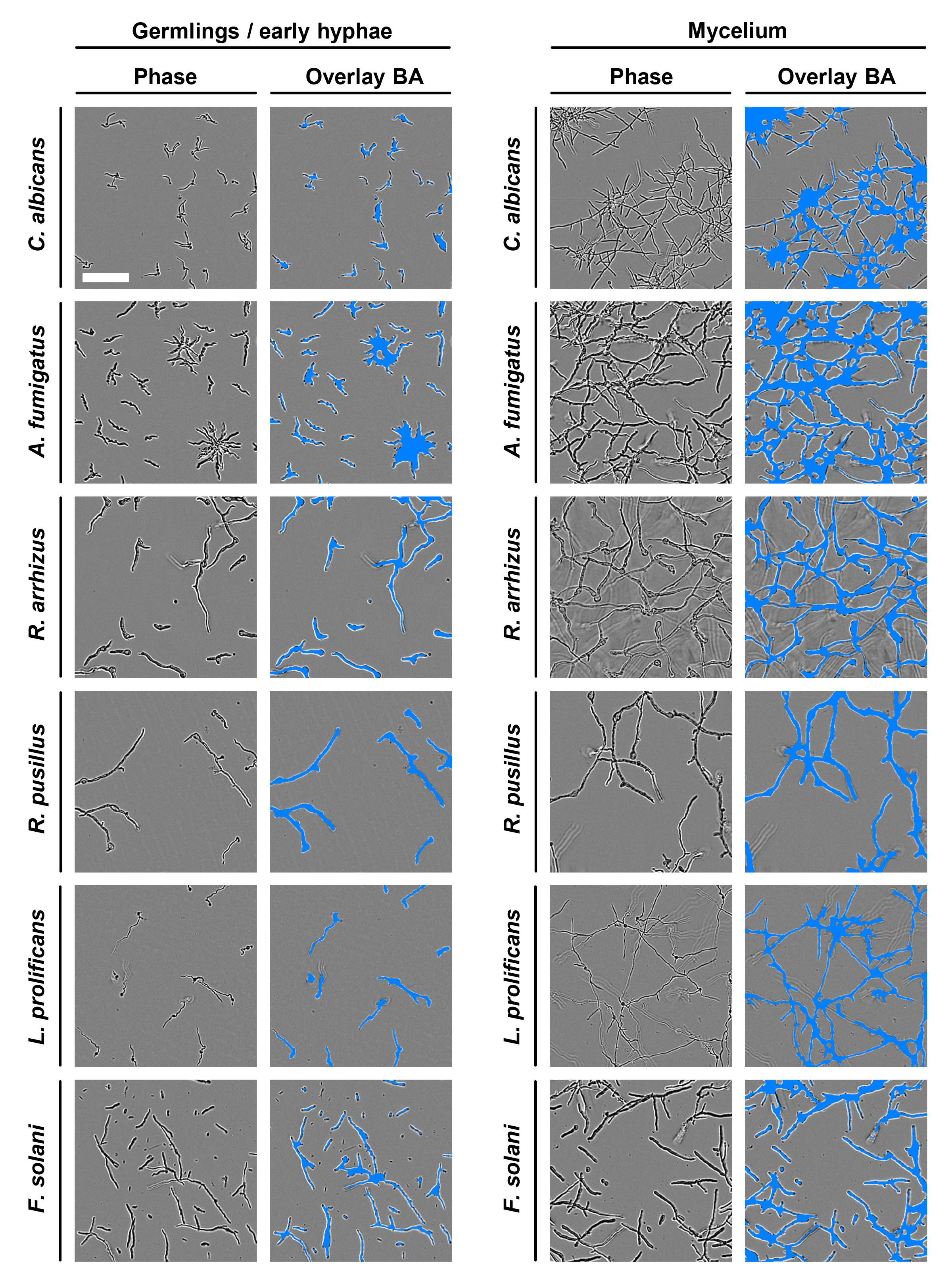

Supplement: FIG S2 [file mBio.00673-19-sf002.tif]

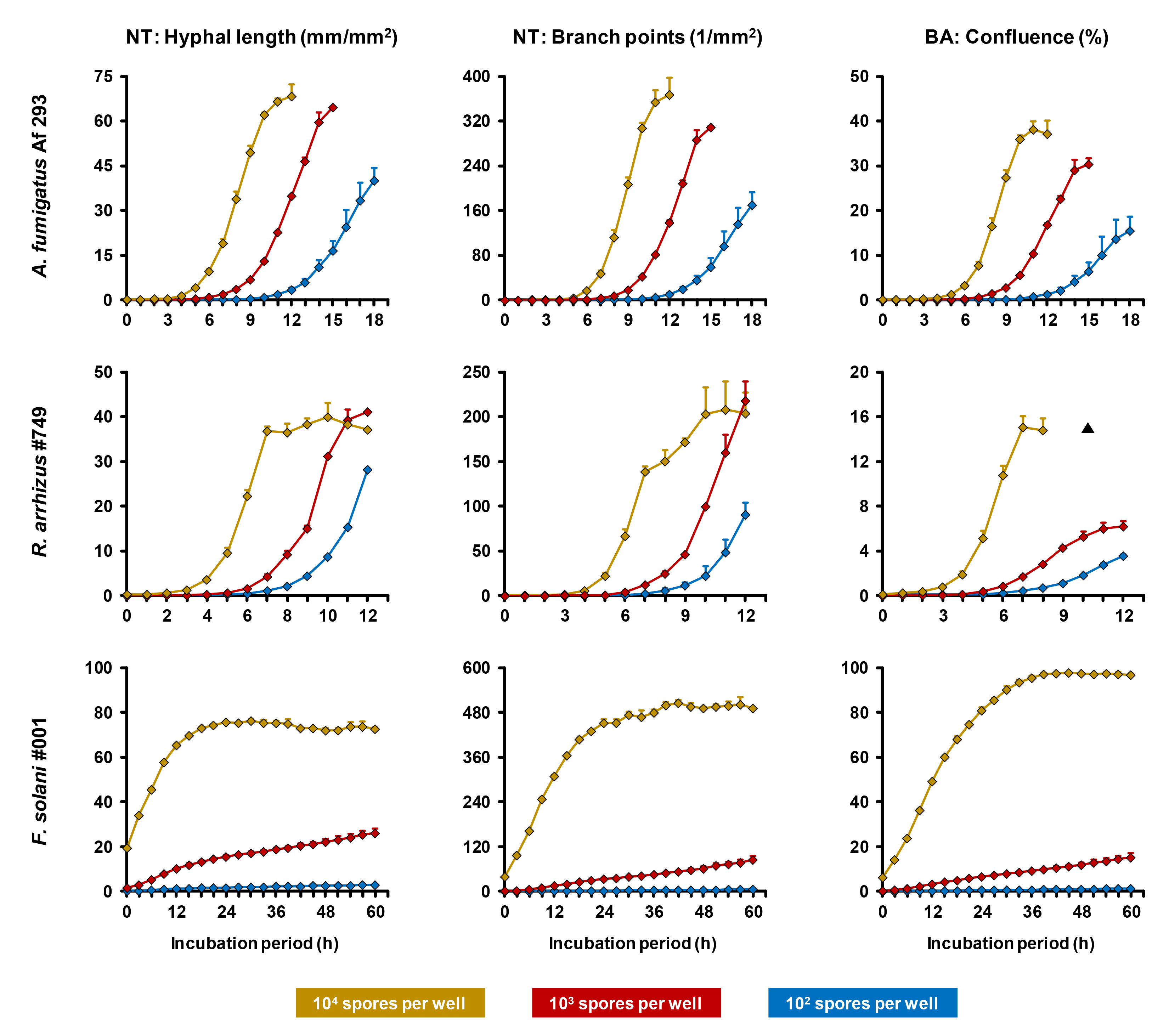

Supplement: FIG S3 [file mBio.00673-19-sf003.tif]
